# Diabetes Self-Management Among Healthcare Providers in King Abdulaziz Medical City, Riyadh: A Cross-Sectional Pilot Study

**DOI:** 10.7759/cureus.18155

**Published:** 2021-09-21

**Authors:** Naif A Alhussein, Moeber M Mahzari, Nader M Aljumaie, Meshari I Alosaimi, Abdulmajeed S Almansouf, Faisal K Alkahtani

**Affiliations:** 1 Medicine, King Saud bin Abdulaziz University for Health Sciences, Riyadh, SAU; 2 Endocrinology, Ministry of National Guard, Riyadh, SAU

**Keywords:** saudi arabia, healthcare providers, type 2 diabetes mellitus, management, control

## Abstract

Background

Diabetes mellitus (DM), a chronic metabolic disease, is a rising global concern with significant social, economic, and health implications. Proper glycemic control is crucial to guarantee protection against these implications such as micro and macrovascular complications. To achieve proper glycemic control, patients' self-management is probably the most essential component, and the development of appropriate self-management behaviors which include medication adherence and lifestyle modifications improves the prognosis and the incidence of DM complications.

Objective

The aim of the study is to examine diabetes self-management and control of diabetic healthcare providers from different specialties working at King Abdulaziz Medical City (KAMC), Riyadh, Saudi Arabia.

Design and setting

This is a cross-sectional pilot study carried out in King Abdulaziz Medical City, Riyadh, Saudi Arabia, using a pre-validated self-administered questionnaire that was “Diabetes Self-Management Questionnaire” (DSMQ), which examined diabetes management and control within the last two months. The questionnaire was distributed to the healthcare providers of all specialties at the site of the study. Correlations and descriptive analyses were carried out using the Statistical Package for Social Science (SPSS) software version 23 (IBM Corp, Armonk, USA).

Results

The total number of participants was 370 healthcare providers (100% response rate). It was found that 26 (7%) of them had diabetes (92.3% of them with type 2 diabetes). The diabetic participants’ mean age was 48.58±7.3 years old. 42.3% were applied medical sciences specialists, with 38.5% having years of experience between 16 to 20 years old. 26.3% were Saudi nationals. The mean HbA1c levels among diabetic patients were 6±1.03%, ranging between (5.1%-9%). There was a significant inverse relationship between each section's scores and total scores with the HbA1c levels (p-value<0.05). Total scores for adherence were significantly higher among the age group (51 to 60) (p-value=0.03) and physicians (p-value=0.035). Dietary control was significantly better among age group (51 to 60) (p-value=0.015), and type 2 diabetes (p-value=0.022). Physician contact was significantly higher in the age group (51 to 60) (p-value=0.027). Physical activity was significantly higher among physicians (p-value=0.030). Blood glucose monitoring was significantly better among the age group (above 60) (p-value=0.026), males (p-value=0.03), and physicians (p-value=0.039).

Conclusion

The findings suggest the glycemic control and adherence to treatment among diabetic healthcare providers in KAMC-Riyadh are adequate the findings suggest the glycemic control and adherence to treatment among diabetic healthcare providers in KAMC-Riyadh are adequate. Future studies with an adequate sample size are essential to assess diabetes self-management and identify if there is any obstacle toward better compliance in healthcare providers.

## Introduction

Diabetes mellitus (DM) is regarded as a complex metabolic disease, that is accompanied by hyperglycemia and affection to multiple organs [1]. Diabetes is usually related to a defect in the metabolism of fats, carbohydrates, and proteins [2]. Moreover, it can result in multiple complications which comprise macro and microvascular complications in addition to neuropathic affection [3].

There are different types of diabetes, though, the most prevalent type is type 2 diabetes mellitus [4]. It is suggested that up to 90% of diabetic patients have type 2 diabetes. Furthermore, the disease is associated with significant morbidity and mortality [5]. Accordingly, it reduces the patient’s quality of life and affects their well-being, hence, diabetes mellitus is a major public health disease [6].

Several therapeutic approaches, lifestyle modifications, risk factors monitoring, and new medications showed effective improvement in the management of diabetes [7]. Additionally, patients who are at risk of diabetes mellitus should be educated about the disease as early as possible to reduce the incidence of the disease and consequently its complications [7]. These educational efforts can significantly reduce the burden on the healthcare systems as well as the economy [8].

The incidence of this metabolic disease is growing worldwide, particularly in developing countries. It is proposed that the number of individuals diagnosed with diabetes is assumed to reach up to 366 million by the year 2030 [9]. Recent reports from the World Health Organization (WHO) demonstrated that the incidence of diabetes mellitus will be doubled in developing countries by the year 2030 [10].

In Saudi Arabia, diabetes mellitus is regarded as a major clinical and public health issue [11]. According to WHO-Diabetes country profiles, 2016, Saudi Arabia reached a 14.4% overall prevalence of diabetes mellitus [12]. In response, Saudi Arabia developed and implemented several policies, guidelines, and monitoring systems to provide high-quality health care [12].

One of the tools developed to assess diabetes self-management among diabetic patients is the Diabetes Self-Management Questionnaire (DSMQ) [13]. It includes several related domains analyzing patients’ behavioral affection in clinical practice. It was utilized among patients with different types of diabetes mellitus [13,14].

There is a scarcity of studies focusing on the control and management of diabetes among healthcare providers in comparison to the general population. Accordingly, the present study aimed to investigate the self-management of diabetes among healthcare providers in KAMC, Riyadh, Saudi Arabia.

## Materials and methods

Study design

A cross-sectional pilot study was conducted at King Abdulaziz Medical City (KAMC) in Riyadh that has been known as a distinguished healthcare organization. KAMC includes surgical and medical units, in-patients' wards, and outpatient clinics of different specialties for a huge patient population in the Kingdom of Saudi Arabia (KSA) at the level of different regions [15].

This study targeted all KAMC-employed healthcare providers of both genders who were diagnosed with DM from all specialties including physicians, dentists, pharmacists, nurses, and applied medical sciences specialists in rehabilitation, respiratory therapy, clinical laboratory, clinical nutrition, radiology, emergency services, etc. It excluded any healthcare provider who was a non-English speaker.

One hundred eighty-eight participants were the minimum recommended sample size as calculated using Raosoft sample size calculator (Raosoft Inc., Seattle, USA) with a margin of error of 5%, 95% confidence level, and Saudi diabetes overall prevalence of 14.4% [12].

Considering this was a cross-sectional pilot study, the sample size was decided to be 10% of the minimum calculated sample size.

Data collection

The study was carried out using a pre-validated self-administered questionnaire that was “Development of the Diabetes Self-Management Questionnaire" (DSMQ) which examined diabetes management and control within the last two months [13]. The approval was obtained from the authors. The distributed questionnaire included three sections. The first section was the informed consent, and the second section was about the participant demographic data (age, gender, nationality, occupation, years of experience, and family history of DM). The participants were then asked if they had DM to continue the third section of the questionnaire that was introduced by three questions (Which type of diabetes do you have? What type of diabetes medications do you take? What was your last Hemoglobin A1c?). The third section was the DSMQ which consisted of sixteen questions on self-care activities related to diabetic patients. These questions were about blood glucose monitoring, dietary control, medication adherence, physician contact, and physical activity. The participants answered the questions using a 4-point Likert scale (0-3). Where 3 was “applies to me very much”, and 0 was “does not apply to me”. The total score was the sum of items that ranged between 0-48. Higher scores indicated more effective self-care. Scores were then factorized 0- 10 points. The questionnaire required about two minutes to be completed. 

The method was convenient sampling where all available participants were approached in their departments and provided face-to-face with the questionnaire hardcopy. The questionnaires were collected once completed. The participants’ data were obtained by trained data collectors who entered, categorized, and arranged them by a pre-designed related Microsoft Excel 97-2003 Worksheet (Microsoft Corporation, Redmond, USA). 

Statistical analyses

The data were coded and analyzed using the Statistical Package for Social Science (SPSS) version 23 (IBM Corp, Armonk, USA). Statistical analysis was executed in the form of frequencies and percentages to represent categorical variables. Additionally, means and standard deviations expressed numerical variables. The comparison of mean scores was done by a one-way ANOVA test, at a level of significance p-value<0.05. Pearson correlation was carried out between HbA1c levels and scores of different sections of the questionnaire. 

Ethical considerations

Research ethics board approval was acquired prior to starting any study procedure. The ethical approval for this study was obtained from the King Abdullah International Medical Research Center (KAIMRC) in Kingdom of Saudi Arabia (IRP No.: SP19/350/R). The patients' identities were kept confidential.

## Results

The study included 370 healthcare providers who participated in the study; of them, 26 healthcare providers were diabetic and were fitting in our inclusion criteria. The whole cohort is described below.

General characters of patients

Of the 26 diabetic responders, 73.1% were females, and 61.5% were in the age group between 41 to 50 years old. The mean age of the patients was 48.58±7.3 years old. As for the medical specialty, 42.3% were applied medical sciences, with 38.5% having years of experience between 16 to 20 years old. 26.3% of the patients were Saudi nationals, as illustrated in Table 1. 

**Table 1:** Demographic data of the responders.

		Non-Diabetic		Diabetic	
		Count	Percent	Count	Percent
Gender	Male	142	41.3	7	26.9
	Female	202	58.7	19	73.1
Age category	20-30	172	50.0	0	0
	31 to 40	113	32.8	3	11.5
	41 to 50	44	12.8	16	61.5
	51 to 60	14	4.1	5	19.2
	More than 60	1	0.3	2	7.7
Occupation	Applied Medical Science	103	29.9	11	42.3
	Dentist	26	7.6	0	0
	Nurse	82	23.8	8	30.8
	Pharmacist	21	6.1	0	0
	Physician	112	32.6	7	26.9
Nationality	Saudi	235	68.3	7	26.9
	Non-Saudi	109	31.7	19	73.1
Years of experience	Less than or equal to 5 years	178	51.7	0	0
	6 to 10 years	76	22.1	0	0
	11 to 15 years	42	12.2	6	23.1
	16 to 20 years	26	7.6	10	38.5
	21 to 25 years	6	1.7	5	19.2
	25 to 30 years	12	3.5	2	7.7
	More than 30 years	4	1.2	3	11.5

Family history

As for the family history of responders, for diabetic patients, 46.2% had more than one first-degree relative with diabetes, while for the non-diabetic responders, 46.8% did not have any first-degree relatives with diabetes, as shown in Table 2.

**Table 2:** Family history.

		Non-Diabetic		Diabetic	
		Count	Percent	Count	Percent
Family history	Brother	5	1.5	1	3.8
	Father	87	25.3	4	15.4
	Mother	49	14.2	6	23.1
	Sister	5	1.5	0	0
	None	161	46.8	3	11.5
	More than one	37	10.7	12	46.2

Description of diabetes mellitus

Diabetic patients were asked about the characters of their disease. 92.3% had type 2 diabetes, while the rest had gestational diabetes (two patients). As for medical treatment, the most common medication used was metformin. Insulin was only used by one patient and was combined with oral agents. Monotherapy was used in 34.6%, while combination therapy was used by 61.5%., as described in Table 3.

**Table 3:** Description of diabetes mellitus.

		Count	Percent
Type of diabetes	Gestational diabetes	2	7.7
	Type 2 diabetes	24	92.3
List of Medication	Monotherapy	9	34.6
	Combination therapy	16	61.5
	Insulin (in combination)	1	3.8

Responses to diabetes self-management questionnaire (DSMQ):

Diabetic patients were asked to answer the validated DSMQ sections, including blood glucose monitoring, physician contact, medication adherence, dietary control, and physical activity. The healthcare providers chose from four Likert choices including (applies to me very much, applies to me to some degree, applies to me to a considerable degree, and does not apply to me); the healthcare providers were then scored such that the best response indicating good practice was given three points, while response indicating poor practice was given zero points. Scores were then factorized such that the total score to be out of 10 points [13]. Higher scores represent higher medication adherence and control of diabetes [13]. Full responses of diabetic patients are shown in Table 4. 

**Table 4:** Responses to diabetes self-management questionnaire (DSMQ).

	Applies to me very much (%)	Applies to me to a considerable degree (%)	Applies to me to some degree (%)	Does not apply to me (%)
Blood glucose monitoring				
I check my blood sugar levels with care and attention.	30.8	50	19.2	0
I record my blood sugar levels regularly (or analyze the value chart with my blood glucose meter).	23.1	34.6	38.5	3.8
I do not check my blood sugar levels frequently enough as would be required for achieving good blood glucose control.	7.7	23.1	30.8	38.5
Physician contact				
I keep all doctors’ appointments recommended for my diabetes treatment.	61.5	26.9	7.7	3.8
I tend to avoid diabetes-related doctors’ appointments.	0	11.5	19.2	69.2
Regarding my diabetes care, i should see my medical practitioner(s) more often.	30.8	34.6	26.9	7.7
Medication adherence				
I take my diabetes medication (e. g., insulin, tablets) as prescribed.	69.2	15.4	11.5	3.8
I tend to forget to take or skip my diabetes medication (e. g. insulin, tablets).	0	19.2	26.9	53.8
My diabetes self-care is poor.	3.8	19.2	26.9	50
Dietary control				
Occasionally i eat lots of sweets or other foods rich in carbohydrates.	15.4	26.9	50	7.7
Sometimes i have real 'food binges' (not triggered by hypoglycemia).	11.5	30.8	46.2	11.5
The food i choose to eat makes it easy to achieve optimal blood sugar levels.	57.7	23.1	15.4	3.8
I strictly follow the dietary recommendations given by my doctor or diabetes specialist.	30.8	34.6	34.6	0
Physical activity				
I do regular physical activity to achieve optimal blood sugar levels.	23.1	42.3	26.9	7.7
I avoid physical activity, although it would improve my diabetes.	0	15.4	30.8	53.8
I tend to skip planned physical activity.	0	19.2	42.3	38.5

Total scores for each section were calculated, and the grand total score was calculated for all the sections. The total scores were then factorized to be out of 10 points. It has been shown that mean scores were above average for the five sections and the total score except for physical activity, as shown in Table 5. 

**Table 5:** Mean scores for each section and the full questionnaire.

	Mean	SD	Minimum score	Maximum Score
Blood glucose Monitoring	5.3	1.9	2	10
Dietary control	8.0	3.2	1	9
Medication adherence	9.6	2.7	5	10
Physician contact	5.1	1.3	1	8
Physical activity	2.7	1.7	0	7
Total score	5.3	1.4	2	8

Correlation between glycated hemoglobin levels and scores of each section

Pearson correlation was performed between HbA1C levels of the patients and their scores for each section (Table 6). It has been shown that there was a significant inverse relationship between the scores of each section and total scores versus the HbA1c levels. The mean HbA1c levels among diabetic patients were 6±1.03%, ranging between (5.1%-9%).

**Table 6:** Correlation between glycated hemoglobin and scores of each section.

	Correlation coefficient	P-value
Total score	-0.161	0.043
Dietary control	-0.009	0.046
Medication adherence	-0.178	0.038
Physical activity	-0.182	0.037
Blood glucose Monitoring	-0.007	0.029
Physician contact	-0.221	0.027

Comparison of mean scores among diabetic patients over different demographic variables

Through one-way ANOVA testing at a level of significance p-value<0.05, mean scores for each section, and the total scores were compared over different demographic variables. It has been shown that total scores were significantly higher among the age group (51 to 60) (p-value=0.03), and physicians (p-value=0.035). Dietary control was significantly better among age group (51 to 60) (p-value=0.015), and type 2 diabetes (p-value=0.022). Physician contact was significantly higher in the age group (51 to 60) (p-value=0.027). Physical activity was significantly higher among physicians (p-value=0.030). Blood glucose monitoring was much better among the age group (above 60) (p-value=0.026), males (p-value=0.03), physicians (p-value=0.039), as shown in Table 7. 

**Table 7:** Comparison of mean scores among diabetic patients over different demographic variables. * P-value is significant. Data presented as mean ± standard deviation.

		Total score	P-value	Dietary control	P-value	Physician contact	P-value	Physical activity	P-value	Blood glucose monitoring	P-value	Medication adherence	P-value
Age group	31 to 40	4.6±1.6	0.03*	2.6±1.5	0.015*	3.6±0.6	0.027*	4±2	0.401	4.6±1.1	0.026*	9.6±2.5	0.051
	41 to 50	2.4±0.6		4.3±1.6		2.5±1.3		2.8±1.5		4.4±1.7		8.3±2.3	
	51 to 60	7.6±1.4		5.2±0.8		3.8±1.7		4±1.8		5.8±1.1		9.8±2.8	
	More than 60	4.5±0.7		4.5±0.7		2.5±0.7		3.5±0.7		6.5±3.5		7.5±2.1	
Gender	Female	3.3±0.8	0.399	4.1±1.7	0.418	2.9±1.4	0.889	3.1±1.6	0.355	4.5±1.6	0.03*	8.6±2.3	0.978
	Male	5.8±1.1		4.7±0.7		2.8±1.6		3.7±1.5		5.8±1.9		8.7±2.8	
Nationality	Saudi	2.7±1	0.541	3.8±0.8	0.369	2.4±0.9	0.287	2.7±1.5	0.322	5.2±1.9	0.495	8.4±1.7	0.744
	Non-Saudi	4.5±0.2		4.4±1.6		3.1±1.5		3.4±1.6		4.7±1.7		8.7±2.6	
Occupation	Applied Medical Science	2.9±0.6	0.035*	4.3±1.7	0.922	2.6±1.5	0.692	2.8±1.7	0.030*	4.8±1.8	0.039*	8.2±2.2	0.632
	Nurse	2.8±0.7		4.1±1.8		3.1±1.3		3.1±1.5		3.8±1.2		8.6±2.6	
	Physician	7.1±0.1		4.4±0.5		3.1±1.4		4±1.3		6.1±1.6		9.4±2.6	
Years of experience	11 to 15 years	6.5±0.5	0.689	4.2±1.9	0.945	3.8±1.7	0.017*	3.8±1.6	0.543	5±1.5	0.610	9.6±2.7	0.721
	16 to 20 years	3.8±0.9		4.3±1.4		3.1±1.4		3.2±1.5		4.7±2		8.5±2.5	
	21 to 25 years	2.6±0.5		4±2		1.8±0.8		2.2±1.5		4.2±1.3		8.4±2.3	
	25 to 30 years	6±0.4		5±1.4		3±1.4		3.5±3.5		5±1.4		9.5±3.5	
	More than 30	4.3±0.6		4.6±0.6		2.3±0.6		3.6±0.6		6.3±2.5		7.3±1.5	
Type of diabetes	Gestational	6±0.2	0.069	2±0.1	0.022*	2±0.1	0.346	3±0.1	0.835	3.0±0.1	0.120	6±0.1	0.104
	Type 2 Diabetes	4.7±0.3		4.5±1.4		3±1.4		3.2±1.6		5.04±1.7		8.9±2.3	

## Discussion

Diabetes mellitus is a leading cause of mortality and morbidity among chronic diseases all over the world [16]. Non-adherence to medications and lifestyle modifications are significant contributors to uncontrolled glycemic levels [17]. Additionally, uncontrolled blood glucose level is a significant risk factor for the occurrence of diabetic complications and mortality compared to controlled glycemic levels [18].

The present study aimed to examine the management and control of type 2 diabetes mellitus among diabetic healthcare providers. The study demonstrated that the prevalence of diabetes among the included cohort of healthcare providers was 7% with a mean age of 48.58±7.3 of which type 2 diabetes mellitus was the most common with 92.3% while the rest of the patients had gestational diabetes. The included patients showed adequate HbA1c control, which was parallel to higher adherence demonstrated by the mean scores of the questionnaire. The mean HbA1c levels among the diabetic patients were 6±1.03%, ranging between (5.1%-9%), while the mean scores for each of the five sections and the total scores were above average. 

Despite these positive results, it is worth mentioning that some of the healthcare providers showed significantly better adherence than others over different sections of the questionnaire. The total scores were significantly higher among the age group (51 to 60) (p-value=0.03), and physicians (p-value=0.035). Dietary control was significantly better among age group (51 to 60) (p-value=0.015), and type 2 diabetes (p-value=0.022). Physician contact was significantly higher in the age group (51 to 60) (p-value=0.027). Physical activity was significantly higher among physicians (p-value=0.030). Blood glucose monitoring was much better among the age group (above 60) (p-value=0.026), males (p-value=0.03), physicians (p-value=0.039). 

Medication adherence and glycemic control have been investigated using the Diabetes Self-Management Questionnaire (DSMQ) in different settings. Liu et al. [19] described the self-management adherence and control of type 2 diabetes using the DSMQ questionnaire in Taiwan patients. By including 192 patients aging above 65 years old, Liu et al. [19] demonstrated that older patients with higher educational levels and above-average scores on the DSMQ questionnaire showed better control of HbA1c.

Similarly, the present study demonstrated that the higher the mean scores in each of the five sections and the total score, the better the control of HbA1c levels. This is reflected by the patients included in the present study who had a mean HbA1c level of 6±1.03%, which lies within the accepted HbA1c level for diabetes control. Furthermore, the included cohort also showed above-average mean scores in all five sections and the total score. 

Another recent study by Ji et al. [20] used the DSMQ questionnaire to examine diabetic control and adherence in the Chinese population. Ji et al. [20] included 207 patients and demonstrated that patients with higher scores were correlated to better control of HbA1c levels. Higher scores were particularly demonstrated in the medication adherence, physical activity, and dietary control section.

Moreover, another study that was carried out in Mexico by Lavalle-Gonzalez et al. [21] that included patients from both type 1 and type 2 diabetes mellitus and used the DSMQ questionnaire showed that patients with better scores were medical providers, with better HbA1c control and fasting blood glucose control [21].

Although the present study did not include patients with type 1 diabetes, the present study supports the findings of Ji et al. [20] and Lavalle-Gonzalez et al. [21], demonstrating a significant correlation between the mean scores and glycemic control. Additionally, all the included subjects were healthcare providers, unlike the sample recruited by Ji et al. [20]. The present study also showed that some demographic variables were significantly affecting the adherence and glycemic control scores, as explained before. 

Nevertheless, it should be noted that the present study suffered from some limitations. Due to the survey nature of this study, the responses of the responders depended mainly on their honesty and subjective opinions, which might affect the reliability of the study. Additionally, the study was carried out in one center in Saudi Arabia, limiting the external validity of the data. Finally, only a small percentage of our cohort had diabetes, which might have affected some variables' statistical significance. These limitations should be considered in any future studies. 

Strengths

This is the first study aimed to assess diabetes self-management among healthcare providers in KSA and discuss different related associations. Based on our experience, approaching the targeted participants through face-to-face allowed permitted the 100% response rate 

Limitations and recommendations

There were some limitations to this study that need to be addressed in any future work. This study attempted to measure the prevalence of DM among the health providers additionally to the primary objectives. Therefore, it was planned to approach all healthcare providers’ (diabetic and non-diabetic participants) from their departments. That has led to minimizing the chance to meet the targeted diabetic participants and lessen the sample size. It should be noted that this was a pilot study to pilot our methodology. It was limited to a single center, and its convenient small sample size was anticipated. Accordingly, the generalizability of its findings will be affected. So, in the upcoming cross-sectional studies where the calculated sample size must be applied, it would be recommended to keep the attention on the primary objective where self-management is the main concern and implement a strategy to approach diabetic participants only through the medical registration department where all employed health providers’ medical records were coded, and their clinic visits were archived.

## Conclusions

The glycemic control and adherence to diabetes management are adequate among diabetic healthcare providers in King Abdulaziz Medical City, Riyadh, Saudi Arabia. This practice reflects good attitudes towards adherence to antidiabetic medications as well as lifestyle modifications. However, some age groups and specialties showed better control levels than others. Accordingly, through the present findings, the research team would recommend performing similar studies with adequate sample size in other to identify any barriers, if present, towards better adherence and control of diabetes among diabetic healthcare providers.
